# Factors influencing behavioral intentions of graduating pharmacy students regarding interprofessional collaboration – a theory-driven qualitative study

**DOI:** 10.1186/s12913-023-10224-0

**Published:** 2023-11-06

**Authors:** Piotr Przymuszała, Sandra Fabianowska, Łucja Zielińska-Tomczak, Magdalena Cerbin-Koczorowska, Ryszard Marciniak

**Affiliations:** 1 Department of Medical Education, Poznan University of Medical Sciences, 7 Rokietnicka St, Poznan, 60-806 Poland; 2 Students’ Scientific Club of Medical Education, Department of Medical Education, Poznan University of Medical Sciences, Poznan, Poland; 3 Edinburgh Medical School: Medical Education, Chancellor’s Building, University of Edinburgh, Edinburgh, Scotland UK

**Keywords:** Pharmacy students, Pharmacists, Interprofessional collaboration, Behavioral intentions, Theory of planned behavior

## Abstract

**Background:**

Interprofessional collaboration enhances the use of competencies of different medical team members. Additionally, pharmacists’ involvement in patient care has been shown to improve their outcomes and seems particularly valuable in chronic diseases. Therefore, due to the aging of society and the increasing prevalence of chronic diseases, efforts are needed to involve pharmacists more broadly in patient care. However, there is a limited understanding of what factors could influence their willingness to participate in an interprofessional care team, especially among pharmacy students only entering the profession. This study aimed to fill these knowledge gaps by exploring graduating pharmacy students’ intentions in this regard guided by the theory of planned behavior.

**Methods:**

We conducted seventeen semi-structured interviews with graduating pharmacy students of Poznan University of Medical Sciences, which were then thematically analyzed by two researchers.

**Results:**

Positive attitudes of respondents, who mentioned the possibility of acquiring new knowledge, skills, and competencies, increased prestige and appreciation of the pharmacist’s profession, a better quality of patient care, and the support and relief offered to other team members as a result of collaboration, seem to strengthen their intentions. However, they also expressed their worries about potential errors and knowledge gaps, which constituted an example of some existing negative feelings. The identified sources of generally mixed social pressure toward the behavior included other pharmacists and pharmacy students, physicians, patients, their relatives and friends, and the decision and policymakers. Finally, their intentions may also be weakened by the mentioned perceived behavioral control aspects, like their level of pharmaceutical and clinical knowledge, skills, and experience, their knowledge of representatives of other professions and collaboration, the atmosphere at their future place of work, co-workers’ potential reluctance to collaborate with them, finding time for collaboration, the existence of incentives and gratification system, and existing legal regulations.

**Conclusions:**

The attitudes of graduating pharmacy students toward interprofessional collaboration seem mostly positive, but their intentions might be weakened by the mixed sense of social pressure and factors decreasing their perceived behavioral control.

**Supplementary Information:**

The online version contains supplementary material available at 10.1186/s12913-023-10224-0.

## Background

Interprofessional care allows for better use of the knowledge and skills of individual representatives of medical professions [1–3]. In fact, the response to the question of how to improve the quality of patient care can be sought in creating medical teams comprising representatives of various medical professions, including pharmacists. Their broader involvement in patient care seems to be especially justified in the case of chronic diseases such as asthma, diabetes, or hypertension [4–9]. This is important given that the aging society and piling up health problems make healthcare systems less efficient in many countries, including Poland, and more and more medical interventions concern people with chronic diseases [10–13]. Studies show that the effectiveness of care for such patients is improved when a pharmacist is included in the team. For example, a systemic review and meta-analyses conducted by Chisholm-Burns et al. [14] revealed positive effects of pharmacists’ involvement in patient care on different outcomes, including therapeutic (e.g., glycated hemoglobin, cholesterol, blood pressure), medication adherence, adverse drug events, or patients’ knowledge and life quality.

There are more and more examples of interprofessional collaboration (IPC), for instance, between pharmacists and physicians across the globe, like the involvement of pharmacists in clinical work in the United States [15], meetings of pharmacists and physicians in the Netherlands to improve pharmacotherapy and establish prescriptions or therapeutic guidelines for the local community [16], or the service offered by Australian pharmacists called ‘*Home Medicines Reviews*’ - participation in reviews aimed at improving not only the effectiveness but also the safety of the treatment [17]. Also, the World Health Organization recognizes the importance of physician-pharmacist collaboration in improving patients’ safety and outcomes or decreasing the incidence of medical errors [1]. However, problems in full acknowledgment of pharmacists’ potential by medical staff members are still visible. For instance, pharmacists and physicians from Canada surveyed by Kelly et al. [18] agreed on the positive effects of collaboration on patient care and expressed willingness to collaborate more. However, they had different opinions on the areas of this collaboration. In their study, physicians had a greater appreciation for pharmacists’ technical duties and were less favorable toward their clinical functions. In Poland, despite examples from the world indicating its benefits for patients and the results of the conducted research, unfortunately, this type of collaboration is still rare and mostly concerned with formal matters [19, 20]. The most common example of the relationship between a physician and a pharmacist is the traditional model of care, where the doctor diagnoses and prescribes treatment, and the pharmacist implements the doctor’s recommendations. The level of cooperation, according to the three-point scale proposed by Bradley et al. [21], can be assessed at the lowest level of isolation. Professions are geographically separated, and the pharmacist’s role is limited to formal activities, while the physician performs a dominant function [19]. However, recently, the Act on the Pharmacist’s Profession was adopted in Poland, which defines the roles of the pharmacist and the forms of collaboration with physicians [22]. Therefore, ongoing legal changes give these two professions opportunities to establish partnerships in patient care.

As in the case of other healthcare professionals, the involvement of pharmacists in specific activities related to healthcare can be attributed and seems conditioned by their willingness or behavioral intention to take a given action [23, 24]. This behavioral intention, according to Ajzen’s Theory of Planned Behavior (TPB) [25, 26], is, in turn, conditioned by certain variables, which can be divided into attitudes toward behavior, subjective norms, and perceived control of the behavior. Moreover, intentions in TPB can be used as proxy measures of behavior, which is particularly useful when we do not have the tools or possibility to assess the actual behavior [27]. In line with the explanations provided by the theory, attitudes are individual opinions about a given activity, beliefs, and expectations regarding a specific action that characterize them. Subjective norms are represented by the perception of peer pressure and what is expected of an individual in a particular context. Perceived behavioral control answers the question of how difficult or easy it is to take a certain action. Considering that these factors may also influence each other, the assessment of the intention to undertake a given behavior should be made as a whole [25, 26]. As Armitage and Conner [28] showed in their meta-analysis, TPB can also be useful in predicting the behaviors of healthcare workers, and as a recent study shows, this can also include pharmacists [29].

Providing theoretical foundations for the study was of particular importance to us, given that the lack thereof is believed to constitute a considerable problem in the area of medical education [30] and the so far limited literature data on Polish pharmacy students’ intentions regarding interprofessional collaboration. Meanwhile, thoroughly examining the situation from the perspective of graduating students with respect to all TPB aspects could provide valuable data on their behavioral intentions, factors affecting them in terms of attitudes, subjective norms, and perceived control of the behavior, as well as, if necessary, how to respond to identified obstacles and prepare adequate countermeasures already at the undergraduate level. The studies conducted so far show rather positive attitudes of pharmacy students toward interprofessional collaboration, which are higher than medical students, for example [31–35]. However, previous research has also suggested that several factors may negatively influence their intentions toward interprofessional collaboration. For example, while pharmacy students from Qatar showed readiness and positive attitudes towards collaboration and even perceived new pharmacy graduates as ‘drivers for change,’ they also admitted that this change was gradual and were concerned about the tendencies of some pharmacists to discourage their collaboration attempts with physicians [36]. Respondents of Wilson et al. [37] admitted to being cautious when paying doctors’ attention to potential drug-related issues, adopting strategies like posing indirect questions or putting notes on charts. The discussed phenomenon can also be traced in a paper, which, even in its title, references the response given by a physician to a pharmacy student after being notified of bacteria resistance to prescribed antibiotics - *“How Dare You Question What I Use to Treat This Patient?”* [38]. Several barriers to pharmacists’ involvement in interprofessional collaboration have also been listed in previous studies like pharmacists’ lack of access to patients’ documentation, organizational aspects, their physical separation from areas of patient care, lack of interprofessional education, lack of time and incentives like salary increases, and pharmacists’ low willingness for changing their practice [35]. In a recent study, Polish pharmacists and physicians also mention similar aspects like the need to find time for collaboration or expectations of additional financial remuneration, guidelines, and legal regulations on the issue, the lack of which was seen as limiting the collaboration possibilities [39].

## Materials and methods

### Aim of the study

In this study, we aim to use TPB to assess the behavioral intentions of graduating pharmacy students toward interprofessional collaboration and the factors influencing them, with particular emphasis on how their experiences so far may have influenced them.

### Study design and setting

The study was conducted using semi-structured interviews with participants based on a thematic guide, which was formulated with the help of dedicated manuals [27, 40] in order to answer the following research questions:

- What are the behavioral intentions of graduating pharmacy students toward interprofessional collaboration?
- What are and what factors influence their attitudes in this regard?
- What are their subjective norms and the sources of social pressure that could influence their behavior?
- What impacts their perceived behavioral control in this aspect in terms of controllability dimensions and self-efficacy?

The thematic guide is presented in Table 1. The interviews were conducted between January and July 2022. The recruitment process for the study was conducted with the use of convenience sampling, and prospective participants were invited by e-mail and social media. The inclusion criteria adopted in the study were participants’ consent and being a last-year pharmacy student of Poznan University of Medical Sciences (PUMS) in the academic year of 2021/2022. The decision to conduct the study on the last year of their education path was dictated by their potentially greater experience accumulated during their studies, translating into their ability to more precisely indicate factors influencing their intentions toward interprofessional collaboration and providing more thorough and comprehensive insights on the topic. Before commencing the study, the principal author subjected its protocol to the opinion of the Bioethical Committee of the Poznan University of Medical Sciences, which decided that the approval was not necessary under Polish law (Decision No. KB – 929/21). Nevertheless, during the study, the authors made efforts to ensure its highest ethical standards following the BERA Guidelines [41]. The invitations to participate informed potential participants about the aims and methods of the study and its anonymous character. The protocol of the study was also once again presented to every participant prior to the interview, along with the information on the study’s aims, voluntary character, the anonymity of collected data, and the possibility of withdrawing from the study at any moment. Informed verbal consent for participation was obtained from all respondents before starting the interviews. For the safety of participants and the interviewer, given the COVID-19 pandemic at the time, all interviews were conducted on MS Teams. To avoid any pressure on participants and to increase the probability that they will express their real opinions on the topic, the interviews were conducted by a pharmacy student (the second author), who was also a member of the Student Scientific Club of Medical Education at our Department. Apart from her, the research team involved the principal author (a physician with a Ph.D. degree), two pharmacists with Ph.D. degrees (the third and fourth authors) as well as a physician and professor of medical sciences (the senior author), who are experienced in qualitative methodology and research in the area of interprofessional education and collaboration. Prior to the study, the second author (interviewer) had a meeting with the first and third authors to prepare her to conduct interviews, which involved explaining the theoretical framework of the study, providing information on qualitative research and how to ask questions, discussing the thematic guide question-by-question, and then a mock session where she was asking the questions from the guide.

**Table 1:** Outline of the thematic guide used during interviews

1. Opening questions: - participants’ understanding of the term interprofessional collaboration (IPC) and their previous experiences
2. Attitudes about IPC: - advantages and positive feelings - disadvantages and negative feelings
3. Subjective Norms about IPC: - approval in the environment and positive social pressure sources - disapproval in the environment and negative social pressure sources
4. Perceived Behavioral Control of IPC: - factors enabling or facilitating the behavior - factors preventing or hindering the behavior
5. Participants’ opinions about IPC in Poland – the situation now, possibilities, and improvements that could be introduced
6. Participants’ readiness for IPC
7. Closing question: - additional topics that participants wanted to raise on the subject

### Study participants

Seventeen interviews with graduating pharmacy students of PUMS were conducted – with ten female and seven male participants. The interviews lasted, on average, 40 min.

### Data analysis

The recorded interview material was encoded and analyzed thematically following the steps described by Braun and Clarke [42]: initial familiarization with data, followed by generation of initial codes, searching for themes, reviewing, defining and naming them, and finally, producing the report. When producing the final report, the themes developed during the study were, for clarity, grouped according to TPB as pertaining to attitudes, subjective norms, and perceived behavioral control. To increase the perspective of the study, two researchers performed the described data analysis process [43]. The point of data saturation was observed after fourteen interviews. The paper was prepared considering the standards for reporting qualitative research [44].

## Results

The themes generated in the course of the study are presented below in accordance with TPB and are additionally summarized in Table 2. The quotations of participants’ words are presented in the text in shortened form in order not to impede the flow of the paper. For readers’ convenience, a more detailed account of the respondents’ quotations with respect to the themes developed during the study is presented in the Additional file 1: Appendix.

**Table 2:** Themes developed during the study grouped in accordance with TPB aspects

Attitudes	Subjective norms	Perceived behavioral control
Possibility to acquire new knowledge, skills, and competencies	Pharmacists and pharmacy students	Respondents’ knowledge, skills, and experience
Increased prestige and appreciation of the profession	Physicians	Limited knowledge of representatives of other professions and collaboration
Better quality of patient care	Patients	The atmosphere at the future place of work and co-workers’ potential reluctance
Support and relief offered to other team members	Relatives and friends	Finding time for collaboration
Worries about potential errors and knowledge gaps	Decision and policymakers	Incentives and gratification system
		Legal regulations

### Attitudes

Among the themes relevant from the point of view of attitudes, we generated the following:

#### Possibility to acquire new knowledge, skills, and competencies

Professional development due to acquiring new knowledge, skills, and competencies was seen as an important advantage of collaboration in the interprofessional team in the future. Respondents’ reflections showed enthusiasm over the possibility of getting to know the specificity of a new workplace, meeting new people and exchanging experiences with them, broadening their horizons, or viewing certain topics from the perspective of another profession. As participants noticed, they can learn from members of other professions, but also other professions can learn from them.

P1: *“I think that for me, expanding knowledge also about things that I, for example, was not taught during studies. I think that I can also teach someone, and someone can teach me something, and it is also cool because then a person develops.”*

The opportunity for professional self-development provided by working in an interprofessional team seemed especially important in the context of the limited motivation in the current conditions.

P12: *“Firstly, it is definitely exciting. Secondly, certainly personal development because at the moment I have a limited need for further education after the studies because I assume that there is no point in further learning on such serious topics - let’s say some pharmacological ones because I will not use it anyway, so I prefer to focus on what new cream came out to give the patient a hint. So really, I flatten, so to speak, my competence, and here, for sure, I would see more sense in personal development.”*

#### Increased prestige and appreciation of the profession

The above-mentioned exchange of professional experience was also viewed as a potential means to increase the prestige of their profession in the eyes of society and members of other healthcare professions. This seemed important in the context of the apparent contrast between their image of the profession and the perceived societal image. The satisfaction of being listened to and appreciated by other team members was also viewed as some form of reward.

P3: *“We, as pharmacists, can also show what knowledge we have at this moment, and we do not limit ourselves to being [the respondent uses the word szufladziarze – an untranslatable pejorative plural noun originating from Polish szuflada (drawer), referring to pharmacists as mere sellers of medicaments], as people often see us […] it is certainly an opportunity to prove ourselves.”*

#### Better quality of patient care

Improvements in the quality of provided healthcare services and better patient care were given as other benefits of the involvement of pharmacists in interprofessional collaboration. Respondents listed many ways in which they could contribute to patient care, including getting involved in patient education, optimization and increased effectiveness of pharmacotherapy, reduction of incidence of side effects, and a bigger chance of catching an error. In this context, in the example of physician-pharmacist collaboration, they often referred to the varying expertise of different healthcare professionals. They saw themselves as drug experts whose knowledge would be valuable for physicians perceived as having only general knowledge of drugs and medicinal substances and their effects on the human organism. This was seen as particularly important in the case of patients taking many different medications, elderly or chronically ill patients, or new drugs introduced to the market.

P12: *“Certainly, increasing the quality of care for the patient because one specialist cannot handle everything. Instead, it would be divided into several people, so the quality would be higher because these people could specialize more, and so higher the probability of catching some error.”*

#### Support and relief offered to other team members

Another benefit of the interprofessional collaboration mentioned by the respondents was the support provided by pharmacists to other team members in their everyday work. It covered the above-mentioned aspect of catching any potential errors and the improvements in the productivity and organization of their work. Pharmacists’ involvement as part of the collaborative practice was also seen as a way to relieve other overburdened healthcare workers like doctors or nurses.

P7: *“I believe that teamwork is crucial nowadays, that one person is not able to do much alone, that it is pointless to assume that one person should be responsible for everything, […] And thanks to this collaboration, we can function better, and people are relieved, too. Then one person is not so mentally burdened.”*

#### Worries about potential errors and knowledge gaps

A negative aspect covered by the respondents revolved around the increased professional responsibility of pharmacists compared to the current situation, which constituted a source of stress in the event of potential errors. They also expressed concerns about being adequately prepared and fear of showing a lack of knowledge or competence in front of members of other professions.

P5: *“We would also take responsibility for this patient, for our opinion, which would be put into practice, well, [patient’s] health and life would depend on us. […] And in such a situation that, for example, I analyzed something wrong, and on this basis, a wrong decision was made, which potentially caused the patient to suffer, it could be hard.”*

P6: *“When someone treats you as a specialist, and you feel like a specialist at least a little bit, there is also this fear that you do not know something, that you will say something wrong, that they will ask you a question about something you realize that you should know, but at the moment you do not know. […] I guess the fact that the team’s expectations will be disappointed is my biggest fear in such teams, where we have several different professions.”*

### Subjective norms

Relevant sources of the peer pressure from the point of view of subjective norms were:

#### Pharmacists and pharmacy students

Current and future representatives of their profession were considered to generally hold positive views about getting involved in interprofessional collaboration due to its influence on the increased prestige of the profession, for instance. These individuals were attributed with such qualities as confidence, perseverance, courage, patience, ambition, motivation, and willingness for self-development, as well as the development of the pharmacist’s profession. The need for communication and teamwork skills was also brought up. Also, students engaged in different organizations (e.g., student self-government, student scientific clubs) during their studies were seen as more prone to collaboration after graduation.

P8: *“I think pharmacists would be happy about that. […] [pharmacists and why?] Well, I think that many pharmacists have such a sense of inferiority because of this hierarchy. And they could praise it as it could be a beginning of something in a better direction - abolishing this hierarchy.”*

However, this positive attitude may not necessarily be shared by all pharmacists and students due to poor previous experiences or low motivation and self-efficacy, for instance. The risk of jealousy among other pharmacists was also raised in the context of respondents’ potential work within the interprofessional team.

P14: *“I think pharmacists as a professional group [could disapprove]. I think that some people, because I do not want to make generalizations, might feel jealous that I, as a pharmacist after studies, suddenly go to some interprofessional team - Why cannot they go because they are also good pharmacists and could be there too? So, I think such jealousy could appear somewhere.”*

#### Physicians

For many respondents, physicians were an important point of reference. However, the opinions of participants on this profession in terms of perceived social pressure groups were mixed. Some respondents believed physicians would be pleased with interprofessional collaboration due to its many benefits, including the exchange of knowledge and skills among the professionals or reduced number of physicians’ duties.

P9: *“I think that some doctors would be pleased because our knowledge in the field of, for example, pharmacokinetics, pharmacology, pharmacotherapy is a bit different and often more detailed than that of doctors. […] doctors have the basis in pharmacology, more in the direction of dosing or treatment, while drug interactions at some cellular or metabolism level are more of our area, so surely this would make doctors happy that they do not have to worry so much about combining different drugs, because we would be on guard of this.”*

On the other hand, other respondents had more pessimistic views on physicians’ opinions. Respondents pointed to the existing hierarchical structure of the healthcare system, physicians’ sense of superiority, fear of losing their competencies, and reluctance to share responsibilities with other professions.

P2: *“It seems to me that, at least at first, physicians would probably be dissatisfied that their competencies are taken away from them, that someone meddles in their work.”*

Negative attitudes of physicians towards interprofessional collaboration with pharmacists were more expected among the members of the older generations.

P1: *“Some doctors, especially the older ones, could have a problem with the fact that someone interferes with their work, what they prescribe, takes away a part of their competence.”*

One respondent also referred to the COVID-19 pandemic as an example of the changing image of pharmacists as a result of broadening their scope of practice.

P9: *“The perception of the world of pharmacists is changing, and, for example, in the era of the pandemic, the profession was very much approved by the environment where we were often simply on the front line and had to lead such a patient by the hand in various diseases.”*

#### Patients

Patients, as main beneficiaries of expected improvements in the quality of healthcare services, were regarded to approve of the behavior. They were seen to be especially fond of reduced waiting time to see the specialist or better care and increased effectiveness of the therapeutic process due to the pharmacist’s involvement. Positive effects, and therefore more positive attitudes, were expected to occur, especially in the case of most vulnerable groups like the elderly, children (or their parents), chronically ill patients, or people taking many different medications.

P1: *“I believe that, first of all, such a statistical patient would probably be the most satisfied with such a solution […] such cooperation would definitely relieve the queues, because let’s face it, how many people are registered for a doctor’s appointment only to extend the prescription, for example, for medicines they take permanently. Why cannot it be done, for example, that the doctor knows the patient and introduces them to the pharmacist? The pharmacist continues the therapy, and, for example, once in a while, [diagnostic] tests are ordered so that the patient can see if everything is fine with them. If they are okay, we are moving on with it, and the doctor can admit other people who, for example, have not been diagnosed yet.”*

However, as they noticed, patients’ attitudes on the topic are conditioned on their awareness of its positive outcomes and pharmacists’ competencies.

P5: *“There is still mistrust among patients in a sense what they [pharmacists] can bring or know if they are not a doctor.”*

#### Relatives and friends

Family members and friends were seen as generally accepting of their choices and wanting the best for them. Therefore, they were seen to have positive views on their development and working in the interprofessional team. However, there were also voices that, for some of their relatives or friends, it may be seen as resigning from safe and comfortable working conditions in a pharmacy for something unknown or uncertain.

P2: *“Probably my grandfather, who has a rather stereotypical view of the work of a pharmacist and sees the pharmacist behind the counter only as a seller, so he would probably be happy if I was in such a team because it would go hand in hand with greater prestige, money and that I am near doctors, so it is as if I were a doctor myself.”*

#### Decision and policymakers

Surprisingly, decision- and policymakers at different levels, including hospital management and government entities, were seen as potentially reluctant towards interprofessional collaboration due to the necessity of finding financial resources for it. As respondents noticed, *“from the economic point of view,”* the benefits of pharmacists’ involvement in the patient care team may be hidden compared to the increased costs of employing more staff members or increasing their salaries. On the other hand, this initial reluctance was predicted to disappear once these benefits became clear. The increased prestige of the healthcare facilities involving pharmacists in the work of interprofessional teams was also raised as a factor potentially increasing the willingness of hospital management bodies.

P3: *“In a society, I do not see who would be bothered by it. Unless who would have to finance it? Because, let’s face it, such cooperation would not be pro bono in the long run. Let’s say the NFZ [National Health Fund – governmental insurer agency] could frown upon it a bit until it would see the benefit, which taking some measures can work for the benefit of the patient.”*

P9: *“Maybe it would also be prestige for the hospital management because, for now, in Poland, such a team is still something exclusive, unusual that would distinguish a given medical unit.”*

In this context, the recent COVID-19 pandemic was seen as potentially increasing the understanding of policymakers toward the broader involvement of pharmacists in patient care.

P3: *“I think the pandemic will oblige the NFZ to make better use of our professional group. Well, the fact that vaccinations have started, in addition to COVID, also the flu.”*

### Perceived behavioral control

Themes describing factors influencing respondents’ perceived behavioral control included:

#### Respondents’ knowledge, skills, and experience

The pharmaceutical knowledge that respondents as future pharmacists have and may bring to the interprofessional team was seen as a factor facilitating their future decision on collaboration.

P1: *“To be honest, I feel prepared. I feel that I gained a lot of knowledge during my studies.”*

However, they also referred to their lack of experience and clinical practice. They saw the need to intensify the practical approach to learning during pharmaceutical studies and provide them with relevant clinical knowledge and skills required for patient care. The possibility of gaining this missing experience and skills during their subsequent work in the profession was also acknowledged by respondents.

P2: *“I do not feel I have the experience needed to work in a team like this. However, after a few years of work, if I gained this experience, I think it could be a very interesting escape from working in a pharmacy.”*

#### Limited knowledge of representatives of other professions and collaboration

Respondents also acknowledged their limited occasions to interact with students of other faculties or learn about other professions, resulting in low knowledge about the responsibilities and competencies of other healthcare professions. As remediation, they suggested a broader introduction of interprofessional education or meetings with members of different professions.

P3: *“First of all, cooperation, learning to cooperate with various professionals already at the stage of studies because this is where you have students of medicine, nursing, physiotherapy in one place. Why not use it?”*

Their knowledge of collaboration opportunities and the actual functioning of interprofessional teams was also limited, which seemed to negatively affect their sense of control over the behavior on the general principle of fear of the unknown. Respondents provided several solutions for this situation, including discussing these issues during the studies as well as propositions possible to implement after graduation, like webinars and online workshops, events promoting interprofessional collaboration, or meetings with pharmacists and other healthcare professionals working in interprofessional teams to listen about their experiences, conferences, and internships.

P2: *“First of all, I think that at the level of studies, some classes, which would primarily present in practice how such a team works, or just a conversation with members of such teams would blow up this fog of mystery covering the work of the interprofessional team. I try to make decisions, as much as possible, aware of what I’m getting myself into […] If I knew better what such cooperation looks like, I could imagine whether something like this is for me.”*

Respondents noticed some interprofessional education opportunities offered by the university, for example, in the form of the existing elective classes or student volunteering during the COVID-19 pandemic. However, they viewed them as insufficient as not all students participated in them. Meanwhile, those who had such occasions for collaboration with representatives of other professions during studies described their positive effects on mutual recognition of its importance and competencies of other team members.

P7: *“I was also volunteering at the beginning of the pandemic. It was a student consultation point […] I was in the group, another pharmacy student and a medical student, and we had the opportunity to cooperate and solve various [patient] cases or problems with patients assigned to us. And you could also see a different point of view, and I also know that the girl from medicine was also impressed by how much we were able to help her select drugs.”*

#### The atmosphere at the future place of work and co-workers’ potential reluctance

The relations among their future co-workers, the attitudes of representatives of other professions, potential conflicts within the team, and their potential low willingness to establish collaboration were also mentioned. In this context, respondents also referred to the existing hierarchical structure of medical professions.

P2: *“If I were to cooperate in such a team, it seems to me that if the relations between the members of the team were warm and friendly, it would be easier to cooperate because it would mean that other team members would not take it personally if one would point out a mistake or suggest some change.”*

#### Finding time for collaboration

Respondents also mentioned the aspect of time. It covered the time and engagement they would have to sacrifice for collaboration, their private lives, and potential difficulties in keeping the work-life balance. It was also noticed in this context that pharmacists’ involvement in the work of interprofessional teams could involve the necessity of shift work, which would impose additional discomfort. Respondents also noticed the busy schedules of members of other professions, who could be relieved thanks to the interprofessional collaboration with pharmacists but may not necessarily realize that yet.

P5: *“I am not sure if something like this would give me satisfaction. It seems to me it requires a lot of commitment, and I do not know if at the moment I can say whether I am ready for such a sacrifice.”*

#### Incentives and gratification system

Respondents also referred to the necessity of introducing a reimbursement system corresponding to the increased tasks and responsibilities associated with working in an interprofessional team. They also believed that inadequate incentives or financial remuneration could decrease pharmacists’ intentions in this regard. As one participant noticed in the example of pharmacists’ involvement in performing vaccinations – COVID-19 vaccinations were better priced, translating into a greater interest in administering them than flu vaccinations.

P7: *“Also, probably some salary, depending on how much it would be […] I think that money would also matter. There is no point in hiding that.”*

Additionally, some motivating factor or incentive for working in an interprofessional team was the prestige associated with it.

P12: *“I think some prestige to it as well, if it was some nice hospital, or I knew that it would be such a prestigious job. That would also motivate me additionally.”*

#### Legal regulations

The need for legal stability and the introduction of appropriate and clear legal regulation was also noticed, especially covering the mutual roles, competencies, and responsibility for potential mistakes.

P7: *“All activities a given profession is supposed to perform in such collaboration should be clearly defined. So, who can what and who should say what because there may be some friction, and the pharmacist can step too much in front of the doctor, the doctor can wash their hands, and so on. So, I think the most important thing would be to develop a good system.”*

## Discussion

According to TPB, attitudes signify the evaluation of the behavior, taking into account positive and negative perceptions connected to it and the predicted outcomes [25]. In our study, respondents saw several advantages and positive outcomes of their involvement in interprofessional collaboration, for example, the possibility of acquiring new knowledge, skills, and competencies. They perceived it as a learning and self-development opportunity enabled by the mutual exchange of experiences or perspectives with other team members. A similar value of interprofessional collaboration was noticed by pharmacy students in Qatar, who saw their previous interprofessional experiences as chances for knowledge exchange between professions and learning about other team members’ contributions [36]. An interprofessional compounding workshop described by Taylor et al. [45] also allowed its participants to share knowledge between disciplines and increase their comprehension and appreciation for the pharmacist’s role. Our respondents also believed that pharmacists’ collaboration within an interprofessional team would lead to increased prestige and appreciation of the profession. This is consistent with previous reports reporting dissatisfaction of Polish pharmacists with the perception of their profession as salespersons instead of consultants on health-related issues and the expectations of pharmacists and pharmacy students to broaden their roles and scope of practice [46, 47]. Better quality of patient care as a result of the interprofessional collaboration was the next advantage noticed by our respondents, which is not surprising given the well-documented potential of pharmacists’ contribution to the interprofessional team in terms of patient outcomes, including enhanced patient satisfaction, safety, self-care skills, or acceptance and cost-effectiveness of therapy as well as reduced number of interactions and errors [33, 34]. This concept was also manifested by pharmacy students in a study by El-Awaisi et al. [36], who referred to patient-centered care as a common goal of all healthcare workers and recognized the role of collaboration between them in the improvement of patient care as well as the healthcare system. Our respondents also covered the topic of the support they would constitute to other team members, indicating they could improve productivity and organization of their work and relieve them from some of their duties. Previous studies demonstrate numerous roles and duties pharmacists can have in today’s healthcare system, including taking blood pressure, blood glucose and cholesterol testing, administering vaccines, disease screening, or fitting health-related devices [48]. However, some of our respondents also expressed concerns about the responsibility for potential errors and showing a lack of knowledge or competencies in front of other healthcare team members. Previous studies also provide accounts of the discomfort of healthcare students, including pharmacy students, when students of other professions possess more extensive knowledge on certain topics [49]. Another study showed that while 85% of community pharmacists regarded a pharmacist to be co-responsible for patient treatment, less of them (67%) attributed them with co-responsibility for the consequences of irrational therapy [50]. In a study by El Hajj et al. [51], 29% of pharmacists were not comfortable with taking risks related to responsibility for patients’ treatment outcomes.

Subjective norms focus on respondents’ perceptions of potential sources of pressure and expectations from other people to perform the behavior or not [25]. For our respondents, one such group was other pharmacists and pharmacy students. Although their views were assessed as rather positive, risks of negative attitudes were also raised, which is consistent with previous literature reports. For example, pharmacy students from Qatar showed readiness and positive attitudes towards collaboration and even perceived new pharmacy graduates as ‘drivers for change.’ However, they also admitted that this change was gradual and were concerned about the tendencies of some pharmacists to discourage their collaboration attempts with physicians [36]. Previous reports also show that pharmacy students demonstrate more positive attitudes towards interprofessional collaboration than medical students, for example [31–35]. Additionally, in a study by Seselja-Perisin et al. [31], pharmacists had more positive attitudes than physicians. In our study, participants’ estimates of physicians’ opinions were mixed, with some indicating positive attitudes due to, e.g., reduced number of duties and others negative ones resulting from the hierarchical structure of the healthcare system. An interesting observation in this context was presented in a study by Denvir and Brewer [38], where pharmacy students expressed concern, based on their negative experiences, that physicians could see their unrequested suggestions on patient management as ‘butting in’ or implied criticism. Also, pharmacy students in Qatar were concerned about the attitudes of other healthcare workers, low appreciation for them, and the hierarchization of the system. Physicians were given as a main example of the profession struggling with the changes in pharmacists’ roles who instead still see them as mere drugs sellers [36]. It is also consistent with the principle of least interest, which assumes that *“those who traditionally have been in a more powerful position are less likely to express eagerness for collaborative relationships with others whom they consider to be lower in the power hierarchy”* [33]. Another study conducted on Polish pharmacy students also showed their low belief in physicians’ interest for increased responsibilities of pharmacists and pharmaceutical care implementation [52]. The pharmacist-physician relationship in Poland has been described as isolated, with both professions rarely interacting directly and having low trust levels in their contacts [34]. In a recent study, Polish physicians and pharmacists describe this isolation as often self-imposed and indicate the existence of mutual reluctance for contact among the professions with the suggested importance of factors like existing stereotypes or fear of being judged by another professional [39]. Meanwhile, the way physicians perceive pharmacists and their roles and competencies was suggested to constitute key determinants of good collaboration between both professions [53]. On the other hand, the attitudes of patients as a group potentially most benefitting from a successful collaboration were mostly assessed positively. This is also supported by previous studies on Polish pharmacy students who believed patients would be interested in the pharmaceutical care implementation [52]. However, they also noticed that their positive attitude might be conditioned on their awareness of the benefits of collaboration. Interestingly, similar reflections also appeared in the case of the Polish decision and policymakers, who were regarded as rather reluctant due to financial reasons unless such collaboration would prove beneficial. Polish pharmacists and physicians also notice rather low interest, expectations and involvement of decision and policymakers in regard to interprofessional collaboration [39].

Perceived behavioral control evaluates whether the person feels capable of conducting the behavior, taking into account their control over it and confidence in their abilities [25]. In our study, respondents referred to their pharmaceutical knowledge but noticed gaps in practical and clinical skills and experience. This seems mirrored by the results of other studies on Polish students. For example, most respondents of Świeczkowski et al. [52] expressed disagreement with the statement that their studies curriculum prepared them for providing pharmaceutical care independently, with the most common examples of gaps including advanced pharmacotherapy and medical knowledge like pathogenesis of diseases. However, it should be noted that their study group was divided in this regard, and more than 30% of respondents agreed with the above-mentioned statement. Another study on Polish pharmacy students also reports division in their responses on the readiness for interprofessional collaboration, with more than 39% doubting their qualifications while nearly 35% expressing readiness for professional relationships with other professionals, which, as authors noticed, seemed to be related to the perceived intensity of promoting such collaboration in their program [34]. Similarly, our respondents noticed the importance of contact occasions with representatives of other professions during their education, which they deemed unsatisfactory, and their limited knowledge about collaboration and representatives of other professions. In a study by Wilson et al. [37], recently graduated pharmacists, physicians, and nurses demonstrated similar gaps in their knowledge of mutual competencies, responsibilities, and expertise with reported uncertainty on *“who to consult and when, and what to consult them about.”* Meanwhile, the development of such knowledge, understanding, and trust in competencies and roles of other professions may be hindered, among others, by inadequate or limited opportunities for interprofessional experiences in educational programs [49, 54]. The observation that gaps in knowledge and qualifications, including knowledge of collaboration possibilities and mutual competencies, may lower the intention to collaborate was also noticed by Polish physicians and pharmacists in another study [39]. A solution is sought in interprofessional education, understood to occur *“when two or more professions learn about, from and with each other to enable effective collaboration and improve health outcomes”* [1]. It is believed to enhance students’ understanding of other professionals’ roles and responsibilities, ease of interaction with students of other faculties, and contribute to increased respect and trust and better interprofessional collaboration [36]. Studies conducted so far show positive effects of interprofessional educational interventions and experiences on students’ collaborative and teamwork behavior, understanding, trust development, perceptions, values, and attitudes in this regard [49, 55–57]. Meanwhile, as our earlier study demonstrates, a simple and effective interprofessional class for students can be implemented without generating high costs, which may be of particular importance in countries or universities with limited financial resources [58]. Although, as the study by El-Awaisi et al. [36] shows, some students can feel that their curriculum is already overloaded and suggest an elective form of these classes, others, as also observed among our respondents, indicate the essentiality of interprofessional education for all students. Moreover, the International Pharmaceutical Federation recognizes the crucial significance of interprofessional education and suggests that it *“should, ideally, involve both future and present healthcare workers, and should begin before registration or licensing and persist through the course of the career via continuing professional development”* [59]. However, it should be noted that implementation of interprofessional education may also meet various challenges that need to be overcome, including different class schedules, the already extensive nature of students’ curricula, different lengths of study programs, assessment of such programs, coordination difficulties, limited resources, decision-makers support or students’ attitudes and involvement [54, 60]. Another important factor related to our respondents’ perceived behavioral control was the atmosphere at the future place of work and the potential low willingness of other healthcare team members to collaborate with pharmacists. This seems to mirror the results of another study where, among barriers to collaboration, Polish pharmacy students mentioned excessive competitiveness among professions, their low respect for each other, and their unwillingness to collaborate [34]. Other indicated barriers pertain to mistrust, hierarchy, professional territoriality, and different engagement levels [61]. Additionally, in a recent survey study, Polish pharmacists identified as barriers to collaboration, among others, the absence of collaboration rules, low willingness, mutual respect and trust among professionals, as well as lack of interprofessional integration during studies, insufficient amount of time and deficient legal regulations [20]. It should be mentioned that the situation is slowly starting to improve with the recent introduction of appropriate regulations on pharmacists’ competencies, like the Act on Pharmacist’s Profession [22], which should enable future collaboration initiatives.

Despite the assets of the study, including exploring the under-studied subject with a robust theoretical framework of TPB, we also acknowledge some of its limitations. Since convenience sampling was used, those agreeing to be interviewed could have stronger views on interprofessional collaboration than the rest of their colleagues. However, to reduce this risk, as well as the risk of them hiding their true opinions, the data collection process was performed by the second author, who was their peer, and assured them multiple times that all their points and opinions were important and valid. Furthermore, as the study was conducted on participants from one medical university in Poland, further research on the topic could be beneficial to examine the situation in other settings.

## Conclusion

With the help of TPB, the presented study explored graduating pharmacy students’ behavioral intentions for interprofessional collaboration in their future professional work. Among their positive attitudes, the following issues were identified: the possibility of acquiring new knowledge, skills, and competencies, increased prestige and appreciation of the pharmacist’s profession, a better quality of patient care, and the support and relief offered to other team members as a result of collaboration. Their negative attitudes revolved around their worries about potential errors and knowledge gaps. As for the sources of social pressure on the behavior in the subjective norms, respondents mentioned other pharmacists and pharmacy students, physicians, patients, their relatives and friends, and the decision and policymakers. Finally, factors facilitating or hindering their behavioral intentions in the context of perceived behavioral control included their pharmaceutical and clinical knowledge, skills, and experience, their knowledge of representatives of other professions and collaboration, the atmosphere at the future place of work, co-workers’ potential reluctance to collaboration with them, finding time for collaboration, the role of incentives and gratification system, and existing legal regulations. As the study shows, despite the mostly positive attitudes of respondents, their intentions for involvement in interprofessional collaboration might be weakened by the mixed sense of subjective norms and factors decreasing perceived behavioral control. Conducting such an exploration of the existing set of affairs can be helpful in preparing and implementing adequate and tailored countermeasures to the current situation.

### Supplementary Information

**Additional file 1: Appendix.**

## Data Availability

The datasets used and/or analyzed during the current study are available from the corresponding author on reasonable request.

## Abbreviations

TPB: Theory of planned behavior
IPC: Interprofessional collaboration
PUMS: Poznan University of Medical Sciences
