# Supplementary material for: Factors influencing behavioral intentions of graduating pharmacy students regarding interprofessional collaboration – a theory-driven qualitative study
Source: BMC Health Serv Res. 2023 Nov 6;23:1207. doi: 10.1186/s12913-023-10224-0 (PMC10626734; doi:10.1186/s12913-023-10224-0)
Supplement: Supplementary file 1 — Additional file 1: Appendix. [file 12913_2023_10224_MOESM1_ESM.pdf]

Appendix. A more detailed account of the respondents' quotes in respect to the themes in the study.

Attitudes

Possibility to acquire new knowledge, skills, and competencies

P1: *"I think that for me, expanding knowledge also about things that I, for example, was not taught during studies. I think that I can also teach someone, and someone can teach me something, and it is also cool because then a person develops. I do not feel like I am standing still. I have my area, and that is all I am interested in, and I will be doing the same thing until I retire. It is just such a breath of fresh air that sometimes you learn things you never thought you would learn, and that is natural and cool."*

P14: *"Well, the first thing that comes to mind is development because it seems to me that if there is a team, then we do not insist on our own [opinion] - that we say what we know, but we also learn from other people like how to act, what to do, it is like future-oriented, such a development. Well, development is the first thing that comes to my mind because everyone has something to say, and it is known that we pharmacists, after graduation, are educated pharmaceutically, but a person, for example, after medical studies, can say something else, which we may not even have heard of before, and so can a person after nursing studies, or after laboratory diagnostic [studies]."*

P12: *"It is definitely exciting firstly, secondly certainly personal development because at the moment I have a limited need for further education after the studies because I assume that there is no point in further learning on such serious topics - let's say some pharmacological ones because I will not use it anyway, so I prefer to focus on what new cream came out to give the patient a hint. So really, I flatten, so to speak, my competence, and here, for sure, I would see more sense in personal development."*

Increased prestige and appreciation of the profession

P3: *"We, as pharmacists, can also show what knowledge we have at this moment, and we do not limit ourselves to being [the respondent uses the word szufladziarze – an untranslatable pejorative plural noun originating from Polish szuflada (drawer), referring to pharmacists as mere sellers of*

medicaments], as people often see us [...] it is certainly an opportunity to prove ourselves."

P6: *"I think the most positive feeling is that you can feel like a specialist, that you have the feeling that someone treats you as a person who knows what they are doing, who already has at least some experience, education, and that is a bit of a reward, I have the impression, for the effort we put into these studies, how much we learned and that finally in such a team, where we have people from a different profession, they treat each other as specialists in their respective field and it is like wow - this is something that I know and about which maybe not that I am better, but I excel in something because I know something."*

Better quality of patient care

P9: *"Often being up to date with these changes with some introduction of drugs into circulation, clinical trials or their results, we are also able to inform each other as medical professions about any such changes, so here definitely faster information flow, avoiding side effects, also optimization of doses for example of chronically ill patients, with kidney failure or liver failure depending on how the drug is metabolized. I think that this knowledge often escapes doctors somewhere because simply with their amount of duties, there is no time to think it over, and then I think we would be a great relief for them if we could check whether this pharmacotherapy, the set of drugs that they want to prescribe to the patient, will bring effect or maybe better something else from the same group of drugs, but a substance with fewer interactions or side effects."*

P12: *"Certainly, increasing the quality of care for the patient because one specialist cannot handle everything. Instead, it would be divided into several people, so the quality would be higher because these people could specialize more, and so higher the probability of catching some error."*

Support and relief offered to other team members

P7: *"I believe that teamwork is crucial nowadays, that one person is not able to do much alone, that it is pointless to assume that one person should be responsible for everything, that it is better to have a team, that in a team everyone can cover something different, that it is important that something is, for example, checked by various specialists, right? For example, let's assume it was done by a doctor*

but checked by a pharmacist, dietitian, physiotherapist, or nurse. Everyone works in a different way and can tick off and catch different things. And thanks to this collaboration, we can function better, and people are relieved, too. Then one person is not so mentally burdened."

P13: *"That some things can be done faster. Because there are several [people]- in the sense that the tasks are divided into smaller ones, it seems to me that it is faster. It is also more efficient, and if it is more efficient, it is more economically viable."*

Worries about potential errors and knowledge gaps

P5: *"We would also take responsibility for this patient, for our opinion, which would be put into practice, well, [patient's] health and life would depend on us. And it would be nice if it were always in favor, but it does not always have to be like this. And in such a situation that, for example, I analyzed something wrong, and on this basis, a wrong decision was made, which potentially caused the patient to suffer, it could be hard in this context."*

P6: *"When someone treats you as a specialist, and you feel like a specialist at least a little bit, there is also this fear that you do not know something, that you will say something wrong, that they will ask you a question about something you realize that you should know, but at the moment you do not know. And well, it is normal that we do not know everything and with a little time and our knowledge, even by searching for some information, we can make up for it but just such a fear that someone already requires something from you, because you have this education, but you will not know, and you will come across as an uneducated person and disappoint someone's expectations. I guess the fact that the team's expectations will be disappointed is my biggest fear in such teams, where we have several different professions. […] It probably goes away with time when you work more in a given field, and it comes with experience, but as you are so fresh out of college, I think that this fear of one's competencies is unfortunately still there."*

Subjective norms

Pharmacists and pharmacy students

P8: *"I think pharmacists would be happy about that. There would certainly be praise for it.*

[pharmacists and why?] Well, I think that many pharmacists have such a sense of inferiority because of this hierarchy. And they could praise it as it could be a beginning of something in a better direction - abolishing this hierarchy."

P6: *"Looking at some internet groups, there would be quite a lot of people saying why pharmacists are entering some teams if we do not know anything. [...] I think most would be happy that there is such a possibility, and they would approach it positively. I am not saying that everyone, but I think that for the majority, this is positive news, for pharmacists as a group, that such opportunities appear."*

P14: *"I think pharmacists as a professional group [could disapprove]. I think that some people, because I do not want to make generalizations, might feel jealous that I, as a pharmacist after studies, suddenly go to some interprofessional team - Why cannot they go because they are also good pharmacists and could be there too? So, I think such jealousy could appear somewhere, and these people would not be happy with it."*

Physicians

P9: *"I think that some doctors would be pleased because our knowledge in the field of, for example, pharmacokinetics, pharmacology, pharmacotherapy is a bit different and often more detailed than that of doctors. [...] doctors have the basis in pharmacology, more in the direction of dosing or treatment, while drug interactions at some cellular or metabolism level are more of our area, so surely this would make doctors happy that they do not have to worry so much about combining different drugs, because we would be on guard of this."*

P2: *"It seems to me that, at least at first, physicians would probably be dissatisfied that their competencies are taken away from them, that someone meddles in their work, that it is suggested to them that they may not know something."*

P1: *"Some doctors, especially the older ones, could have a problem with the fact that someone interferes with their work, what they prescribe, takes away a part of their competence [...] Looking at someone who has big experience, does not work clinically, but, for example, has their private office and sees a patient for 15 minutes, takes I do not know 200 [PLN] for it and writes out a prescription*

- well, let's not kid ourselves, but I suspect that they would be moderately happy that someone wants to interfere with this prescription because it could undermine their authority.”

P9: *„Some professions are less appreciated than others, and therefore, today, one side always prevails and believes that their opinion is the truest and correct and does not allow other groups to express their opinion here. However, the young generations are already a bit different. The perception of the world of pharmacists is changing, and, for example, in the era of a pandemic, the profession was very much approved by the environment where we were often simply on the front line and had to lead such a patient by the hand in various diseases.”*

Patients

P1: *“I believe that, first of all, such a statistical patient would probably be the most satisfied with such a solution because even the feeling that someone is looking at you not as a number, but as an individual person [...] also because such cooperation would definitely relieve the queues, because let's face it, how many people are registered for a doctor's appointment only to extend the prescription, for example, for medicines they take permanently. Why cannot it be done, for example, that the doctor knows the patient and introduces them to the pharmacist? The pharmacist continues the therapy, and, for example, once in a while, [diagnostic] tests are ordered so that the patient can see if everything is fine with them. If they are okay, we are moving on with it, and the doctor can admit other people who, for example, have not been diagnosed yet.”*

P5: *“There is still mistrust among patients in a sense what they [pharmacists] can bring or know if they are not a doctor.”*

Relatives and friends

P2: *“Probably my grandfather, who has a rather stereotypical view of the work of a pharmacist and sees the pharmacist behind the counter only as a seller, so he would probably be happy if I was in such a team because it would go hand in hand with greater prestige, money and that I am near doctors, so it is as if I were a doctor myself.”*

P6: *“When it comes to the family, they would also be happy that I would work in such teams. Although*

I do not know what it would be like in my case, sometimes I have the impression that the work of a pharmacist in a pharmacy is such a main goal for older people - <wow you are in a pharmacy> [...] I do not know how my grandmother would react, whether she would not consider it a bit of the decline of my profession actually [...] <You could have a peaceful job in a pharmacy, and you come up with something else? [...] I mean, will you get any money out of it?>"

Decision and policymakers

P3: *"In a society, I do not see who would be bothered by it. Unless who would have to finance it? Because, let's face it, such cooperation would not be pro bono in the long run. Let's say the NFZ [National Health Fund – governmental insurer agency] could frown upon it a bit until it would see the benefit, which taking some measures can work for the benefit of the patient."*

P9: *"Maybe it would also be prestige for the hospital management because, for now, in Poland, such a team is still something exclusive, unusual that would distinguish a given medical unit. Of course, it is known that there are costs and various issues connected to it, but as the research on the West showed, the inclusion of a pharmacist in the team often brings better therapy results, the patient is hospitalized for a shorter time, there are fewer side effects, and at the same time it saves money, and even the payment for such a pharmacist is often lower than the costs that were related to long hospitalization."*

P3: *"I think the pandemic will oblige the NFZ to make better use of our professional group. Well, the fact that vaccinations have started, in addition to COVID, also the flu."*

Perceived behavioral control

Respondents' knowledge, skills, and experience

P1: *"To be honest, I feel prepared. I feel that I gained a lot of knowledge during my studies. I did not necessarily always want to acquire it because it was real torture to learn all these things, but it stays in the head."*

P2: *"At the moment, I do not feel like I would be able to undertake such cooperation. I do not feel that I am properly educated. Maybe not properly educated - I do not feel I have the experience needed*

to work in a team like this. However, after a few years of work, if I gained this experience, I think it could be a very interesting escape from working in a pharmacy."

Limited knowledge of representatives of other professions and collaboration

P3: *"First of all, cooperation, learning to cooperate with various professionals already at the stage of studies because this is where you have students of medicine, nursing, physiotherapy in one place. Why not use it?"*

P7: *"We did not have such classes that would prepare us for what a doctor, a dietician knows. It should be systematized for what, who is responsible, and whom we should ask about what, right? So, the doctors know they can ask me about this and that, but they have no right to demand this and this and this from me. […] I should also know who to ask about something, what to ask the nurse, what to ask the doctor."*

P2: *"First of all, I think that at the level of studies, some classes, which would primarily present in practice how such a team works, or just a conversation with members of such teams would blow up this fog of mystery covering the work of the interprofessional team. I try to make decisions, as much as possible, aware of what I am getting myself into more or less, and even if something sounded interesting or seemed an interesting challenge to me, it could also be that I would not decide on that because I would not know what this job would be all about. It varies, whether it is because of some fear, uncertainty, or I do not want to end up in a situation, let's say, worse than the current one. […] If I knew better what such cooperation looks like, I could imagine whether something like this is for me."*

P7: *"I was also volunteering at the beginning of the pandemic. It was a student consultation point […], and we had patients […] I was in the group, another pharmacy student and a medical student, and we had the opportunity to cooperate and solve various [patient] cases or problems with patients assigned to us. And you could also see a different point of view, and I also know that the girl from medicine was also impressed by how much we were able to help her select drugs."*

The atmosphere at the future place of work and co-workers' potential reluctance

P2: *"If I were to cooperate in such a team, it seems to me that if the relations between the members of the team were warm and friendly, it would be easier to cooperate because it would mean that other team members would not take it personally if one would point out a mistake or suggest some change. [...] I think that such friendly relations would certainly improve cooperation and make it more satisfying and one could fulfill themselves in it because, after all, no matter how great the job is, if the co-workers and the management are, excuse me, a pain in the ass, then a person in a given job will burn out quite quickly."*

P9: *„First of all, the lack of willingness to cooperate with another profession, so if we expressed such a willingness, but the other party considered that we are not worthy or has no desire or willingness to talk, to discuss something with us or to analyze it together, then I would definitely get discouraged."*

Finding time for collaboration

P5: *"I am not sure if something like this would give me satisfaction. It seems to me it requires a lot of commitment, and I do not know if at the moment I can say whether I am ready for such a sacrifice."*

P7: *"It is about the issue of time. Are there enough doctors? So, they would have time for such meetings in the morning, for example, to discuss the patients together, because there is usually no time for all this. Because there is too little staff and there is just no time to focus on this. And I feel that spending more time collaborating could save some time, that sometimes we want to do too much by ourselves [...] I think not everyone is aware of the fact that cooperation could relieve a person, and that is why it is so important."*

Incentives and gratification system

P7: *"Also, probably some salary, depending on how much it would be [...] I think that money would also matter. There is no point in hiding that."*

P12: *"Financial issues, because we have not talked about it yet, that also the way it would be financed because let's face it if it were financed in the same way as in hospitals, doctors earn little money although lately slightly better. But let's say that a pharmacist would not earn as much as a doctor, so less. And now the question is whether I want to take on such a difficult task for money, perhaps worse*

than I would get in a pharmacy because the pharmacy pays quite well now, and the truth is that, well, the responsibility is incomparable because work at the pharmacy well, it is just, so to speak, easier compared to something like this."

P12: *"I think some prestige to it as well, if it was some nice hospital, or I would know that it would be such a prestigious job. That would also motivate me additionally."*

Legal regulations

P7: *"All activities a given profession is supposed to perform in such collaboration should be clearly defined. So, who can what and who should say what because there may be some friction, and the pharmacist can step too much in front of the doctor, the doctor can wash their hands, and so on. So, I think the most important thing would be to develop a good system."*

P14: *"Well, I think I would be scared at first - how will it all be regulated by law? Or if there were frequent changes to these legal regulations, then if I heard that, for example, there was some change somewhere, something was changed from above, then I would feel so insecure that all the time something is changing, and if I take this job, then it can still change. It can change for the better, but it can also change for the worse."*
